# The human microglial HMC3 cell line: where do we stand? A systematic literature review

**DOI:** 10.1186/s12974-018-1288-0

**Published:** 2018-09-10

**Authors:** Cinzia Dello Russo, Natalia Cappoli, Isabella Coletta, Daniele Mezzogori, Fabiola Paciello, Giacomo Pozzoli, Pierluigi Navarra, Alessandra Battaglia

**Affiliations:** 1 Institute of Pharmacology, Università Cattolica del S. Cuore, L.go F Vito 1, 00168 Rome, Italy; 2 Pharmacology Unit, Fondazione Policlinico Universitario A. Gemelli IRCCS, Rome, Italy; 3 Angelini RR&D (Research, Regulatory & Development) - Angelini S.p.A., Rome, Italy; 4 Institute of Human Physiology, Università Cattolica del S. Cuore, Rome, Italy; 5 Institute of Otolaryngology, Università Cattolica del S. Cuore, Rome, Italy; 6 Immunology Laboratory, Department of Oncological Gynecology, Università Cattolica del S. Cuore, Rome, Italy

**Keywords:** Human microglial cell line, HMC3, HMC-3, CHME3, CHME-3, CHME-5, C13-NJ, Molecular phenotype, Molecular signature, Functional properties, IL-6, Chemokines, Free oxygen radicals

## Abstract

Microglia, unique myeloid cells residing in the brain parenchyma, represent the first line of immune defense within the central nervous system. In addition to their immune functions, microglial cells play an important role in other cerebral processes, including the regulation of synaptic architecture and neurogenesis. Chronic microglial activation is regarded as detrimental, and it is considered a pathogenic mechanism common to several neurological disorders. Microglial activation and function have been extensively studied in rodent experimental models, whereas the characterization of human cells has been limited due to the restricted availability of primary sources of human microglia. To overcome this problem, human immortalized microglial cell lines have been developed. The human microglial clone 3 cell line, HMC3, was established in 1995, through SV40-dependent immortalization of human embryonic microglial cells. It has been recently authenticated by the American Type Culture Collection (ATCC®) and distributed under the name of HMC3 (ATCC®CRL-3304). The HMC3 cells have been used in six research studies, two of which also indicated by ATCC® as reference articles. However, a more accurate literature revision suggests that clone 3 was initially distributed under the name of CHME3. In this regard, several studies have been published, thus contributing to a more extensive characterization of this cell line. Remarkably, the same cell line has been used in different laboratories with other denominations, i.e., CHME-5 cells and C13-NJ cells. In view of the fact that “being now authenticated by ATCC®” may imply a wider distribution of the cells, we aimed at reviewing data obtained with the human microglia cell line clone 3, making the readers aware of this complicated nomenclature. In addition, we also included original data, generated in our laboratory with the HMC3 (ATCC®CRL-3304) cells, providing information on the current state of the culture together with supplementary details on the culturing procedures to obtain and maintain viable cells.

## Background

Microglial cells are unique myeloid cells residing in the parenchyma of the healthy central nervous system (CNS). These cells arise from erythro-myeloid precursors in the yolk sac and enter the brain early during development. Although sharing a common lineage with monocyte-derived macrophages, microglia retain unique molecular signatures [1, 2] and acquire specific functional properties which make them substantially different from other populations of myeloid cells present in the brain (i.e., perivascular macrophages, meningeal macrophages, and choroid plexus macrophages) [3]. As the resident immune cells of the brain parenchyma, microglia are constantly monitoring the CNS microenvironment, being able to detect extracellular changes and become rapidly activated in response to different noxious stimuli (effector function). This activity is important in the regulation of brain homeostasis during development and in the adult brain, both in physiological conditions as well as pathology [4]. In addition to their immune functions, microglial cells seem to exert an important role in other cerebral processes, including the regulation of synaptic architecture [5–8] and neurogenesis [9–11], to name a few. Microglia can indeed produce several mediators, among which cytokines (with both pro- and anti-inflammatory activities) and chemokines, but also growth factors and neurotrophins. In addition, they can perform phagocytosis and produce reactive oxygen and nitrogen species, including nitric oxide (NO) mainly produced by the upregulation of the inducible form of NO synthase (iNOS, also known as NOS2). Chronic microglial activation seems to be a common pathogenic mechanism underlying several neurological disorders [12, 13]; therefore, studies on the regulatory mechanisms of microglial activation are important for a large spectrum of cerebral diseases. Accordingly, numerous experimental models have been established to address the issue.

**Fig. 1:**
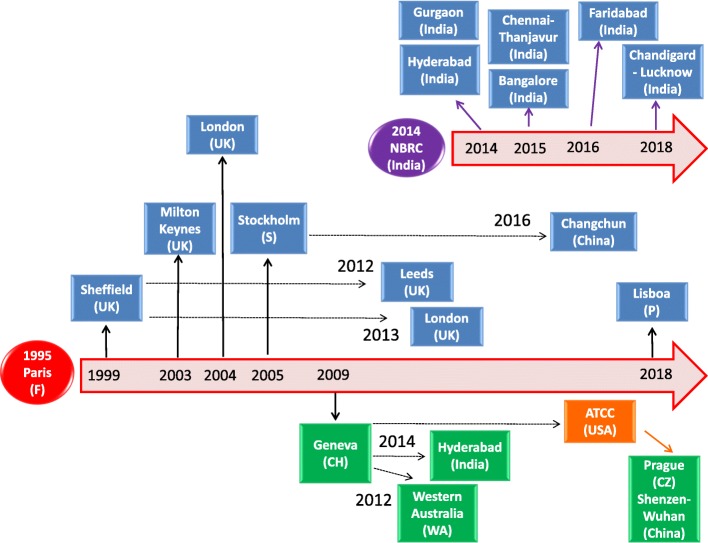
Historical reconstruction of the distribution process of the human microglial clone 3 cell line. The human microglial clone 3 cell line was developed in the laboratory of Prof. M Tardieu, Paris, in 1995 (red circle). As shown in the picture, clone 3 has been distributed worldwide, with the acronym of CHME3 cells (blue boxes) or HMC3 cells (green boxes). Distribution followed two main pathways, either directly from Prof. Tardieu’s laboratory (black thick arrows) or indirectly by the first recipient laboratory (black dotted arrows). A second main distributor of the CHME3 cell line is the laboratory of Prof. A Basu, National Brain Research Centre (NBRC), India (purple circle). Since 2014, this laboratory appears to be the main distributor of the CHME3 cells in India. However, we could not trace on the timeline when the cell line was transferred from the laboratory of Prof. Tardieu to NBRC. In addition, we identified several studies (not reported in the schematic), in which the CHME3 cells were used without any indication of the source, and one study in which the cell line was provided by an Academic institution without any link to published data. In 2016, the HMC3 cells were transferred to ATCC®, USA (orange box) and authenticated and distributed under the catalog designation of HMC3 (ATCC®CRL-3304)

In vitro, microglial functions have been extensively characterized using rodent cultures of microglial cells, either rat primary cultures derived from brain cortices of 1- to 2-day-old newborn animals [14] or immortalized murine cell lines [15]. However, rodent microglia display important biochemical and pharmacological differences compared to human microglia [16]. On the other hand, microglia research using human cells has been limited due to the restricted availability of primary sources of human microglia, including aborted fetal tissue, biopsies from epileptic patients, normal tissue from brain tumor excisions, or postmortem brain tissue. In this respect, human microglial cell lines allow to overcome this problem and can be considered a valuable experimental model. Several cell lines have been generated in different laboratories [17, 18], including more recent lines derived from adult brain tissue [19]. All these cell lines were developed through SV40 immortalization of human primary microglial cells, whereas a fraction of SV40-immortalized adult microglial cells were further engineered to express the human telomerase reverse transcriptase (hTERT) and reduce the proliferation rate [19]. To the best of our knowledge, two cell lines are commercially available, the human microglial clone 3 cell line, HMC3 [17, 20–22] and the “Immortalized Human Microglia - SV40”, developed and distributed by Applied Biological Materials (Vancouver, Canada) [23–28]. The HMC3 has been recently authenticated by the American Type Culture Collection (ATCC®), a leading nonprofit organization in the authentication and distribution of biologic material, microorganisms, and standards. The cell line is distributed by ATCC® under the catalog designation of HMC3 (ATCC®CRL-3304). Interestingly, a PubMed search (database accessed on June 4, 2018), using (HMC3) OR (HMC-3) as keywords retrieved 24 papers, among which only seven are indeed articles related to the human microglial HMC3 cell line, i.e., six original studies [20, 21, 29–32] and one review article [22]. Two of these original papers are also included in the reference list reported in the HMC3 (ATCC®CRL-3304) cell line’s data sheet [20, 21]. However, a more accurate literature revision would suggest that clone 3 circulated among different laboratories also under the name of CHME3 microglial cells. In fact, a PubMed search including (CHME3) OR (CHME-3) as keywords allowed us to identify 21 additional articles, published between 1999 and 2018. All these studies employed the CHME3 cell line, thus contributing to a more extensive characterization of the human microglial clone 3. In Fig. 1, it is reported a historical reconstruction of the distribution process of this cell line across different laboratories, based on published articles. Remarkably, the same cell line has been used in several laboratories with other denominations, i.e., C13-NJ cells and CHME-5 cells. Since “being now authenticated by ATCC®” may imply a wider distribution of the cells, we aimed at reviewing data obtained with the human microglia cell line clone 3, making the readers aware of this complicated denomination. In addition, we also included original data, generated in our laboratory with the HMC3 (ATCC®CRL-3304) cell line, providing information on the current state of the culture together with additional details on the culturing procedures to obtain and maintain viable cells. We believe that a systematic revision of this literature can be regarded as an important starting point for researchers planning to use this cell line in their experimental paradigms.

## The human microglial HMC3 cell line

The human microglial clone 3 cell line, HMC3 [20], was established in the laboratory of Prof. Tardieu in 1995, through SV40-dependent immortalization of human microglial cells [17]. The detailed methodology for the preparation of primary cultures of human microglial cells was previously published [33]. Briefly, microglial cells were isolated by circular shaking from primary mixed cultures of human spinal cord and cortical cells, derived from 8-to 12-week-old embryos and kept in vitro for 10–15 days. Microglial cells were described as slowly diving cells, able to reach confluency in 8–12 days after seeding. The cells showed a complex morphology, with different shapes, a vacuolated cytoplasm, and short processes. In addition, microglial cells were stained positive for several myeloid specific markers, including cluster of differentiation (CD)68, CD11b, and CD14. Interestingly, for the detection of CD68, different antibodies were used, i.e., clones Ki-M7 and Ki-M6 from Behring (Rueil-Malmaison, France) and clone EBM/11 from Dako Corporation (Santa Barbara, CA). More importantly, quantification of CD68 expression varied with the primary antibody used. At in vitro day 1, 83.3 ± 2.9 (± SD) % were detected as CD68 Ki-M7-positive cells, whereas only 33.5 ± 12% were CD68 EBM/11 positive and 18 ± 2.8% CD68 Ki-M6 positive. In addition, primary human microglial cells tended to lose the positivity for CD68 Ki-M6 and EBM/11 during in vitro culturing. Similarly, the expression of CD14 (34 ± 12% of positive cells at in vitro day 1) disappeared after 10 days in culture (< 1% positive cells). Microglial cells were virtually negative for class II major histocompatibility complex (MHCII, 4 ± 4% of positive cells at in vitro day 1, < 1% after 10 days in culture) and CD4 antigen expression. Conversely, most of the cells steadily displayed nonspecific esterase activity (NSE, 90% positive cells at day 1 *vs* 81 ± 1% at day 10) and were able to phagocytize zymosan particles (97% at day 1 *vs* 81 ± 1% at day 10) [33].

Immortalized microglial cells were generated by transfection of the SV40 T antigen in primary human microglial cultures, derived from 8- to 10-week old embryos. Several clones of immortalized cells were isolated, albeit clonality could not be totally confirmed due to inability of the cells to grow at very low density [17]. It should also be pointed out that primary CNS cultures are not necessarily restricted to parenchymal microglia, and other myeloid populations may be present in these cultures, possibly contributing to the culture heterogeneity. Immortalized cells acquired rapid growth capacity (with doubling times ranging between 24 and 48 h) and retained most of the phenotypical and morphological properties of the primary microglial cell source, except for a higher percentage of CD68 EBM/11-positive cells and lower phagocytic activity. Antigenic expression was confirmed to be stable for 35 passages in vitro (data not shown). As summarized in Table 1, the human microglial clone 3 (HMC3 cells) was originally characterized as NSE, CD68, and CD11b positive (80–90%), and CD14, MHCII, CD4 negative under basal conditions [17]. However, the expression level of MHCII increased in response to treatment with human recombinant interferon-γ (IFNγ, 100 U/ml for 18 h; Boeringher-Mannheim, Mayland France) (Table 1). The percentage of MHCII-positive cells (43 ± 10%, ± SD) was higher in HMC3 cells in comparison to other clones (4–13% in clones 1, 2, and 4) and closer to what observed in primary cultures (50%) after stimulation with IFNγ. All the immortalized cells were negative for the specific astrocyte marker, glial fibrillary acidic protein (GFAP), and for the neuronal neurofilament staining (NF70KD) (Table 1). At a functional level, immortalized cells produced and released sizable amounts of interleukin (IL)-6 under basal conditions (Table 2). Interestingly, the HMC3 cells secreted higher amounts in comparison to the other clones [17]. Unfortunately, a direct comparison with primary microglial cells was not included in the paper, and it is difficult to extrapolate from a previous study [34], in which a biological assay was employed to measure the cytokine’s production in place of the enzyme-linked immunosorbent assay (ELISA) adopted later. However, in all the immortalized microglial clones, including the HMC3 cells, basal production of IL-6 was consistently increased by 24-h treatments with human recombinant IL-1α (10 U/ml, Boeringher-Mannheim) or by lipopolysaccharide (LPS) from *Salmonella typhimurium* (Sigma; 10 μg/ml) (Table 2). Again, a direct comparison with primary microglial cultures appears difficult due to substantial differences in the amount of IL-1α/LPS used for the stimulation and the assay employed to assess IL-6 production. However, it seems that the immortalized cell lines were less responsive to LPS in comparison to primary cultures [17, 34]. Similarly to primary cells, all the immortalized microglial cell clones were unable to produce tumor necrosis α (TNFα, data not shown), neither spontaneously nor after pro-inflammatory activation [17]. The production of TNFα was evaluated with a biological assay. Interestingly, lack of TNFα production and CD14 expression was considered a specific property of human embryonic microglia.

**Table 1:** Antigenic profile of the human microglial clone 3 cell line

Markers	Original characterization		CHME3 cells		HMC3 cells		References
	Resting	Stimulated	Resting	Stimulated	Resting	Stimulated	
Myeloid Markers							
CD68 Ki-M7	+++						[17]
CD68 Ki-M6	–						[17]
CD68 EBM/11	++		+				[17, 35]
CD68			+++		−/++	IFNγ: ↑	[20, 29, 38]
CD11b	+++		+ (and also at mRNA level)	Aβ_1–42_: ↑	–	IFNγ: ↑	[17, 20, 29, 39, 40]
CD45					+		[29]
IBA1					+		[20, 29]
MHCII	–	IFNγ: ↑	+		−/+	IFNγ: ↑	[17, 20, 29, 37, 39]
CD14	–				+		[17, 20, 29]
MCSF-R			+ (western immunoblot)				[41]
TLR1			+				[40]
TLR 2 and 6			+++/++	HCV NS3 protein: ↑			[40]
Polarization markers							
CD40			+/++	Aβ_1–42_: ↓/↑			[37, 39, 43]
CD80			+				[37]
CD86			+	Aβ_1–42_: no effect			[37, 39, 43]
CD163			+	Aβ_1–42_: no effect			[37, 43]
CD206			+	Aβ_1–42_: no effect			[37, 43]
Chemokine receptors							
CCR1					+		[29]
CCR2			–		+		[29, 38]
CCR3			++ (MFI)	TNFα: ↑ IFNγ: ↑			[38]
CCR4					++		[29]
CCR5			+		+++		[29, 38]
CCR10			+ (mRNA level)	TNFα:↑ (mRNA level)			[38]
CXCR1			++ (MFI)		++		[29, 38]
CXCR3			+++ (MFI)	TNFα: slight ↑ IFNγ: slight↑	+++		[29, 38]
CX3CR1					+++		[29]
Other markers							
CD4	–						[17]
NSE	+++						[17]
GFAP	–		- (including at mRNA level)		–		[17, 20, 35, 40]
NF70KD	–						[17]
Α7nAcR			+				[44]

Resting conditions: +, 1–33% of the cell population (or a positive immunocytochemistry); ++, 34–66%; +++, > 66%; −, < 1%. Inflammatory stimuli: ↑, upregulation; ↓, downregulation. MFI, mean fluorescence intensity

**Table 2:** Production of cytokines, chemokines, and other inflammatory mediators

Markers	Original characterization		CHME3 cells		HMC3 cells		References
	Resting	Stimulated	Resting	Stimulated	Resting	Stimulated	
Pro-inflammatory molecules							
IL-6	1553 ± 142	IL-1α: > 2500 LPS: 2110 ± 111	20–950/sizable amount	LPS: 2–4 fold ↑ IL-1β: 50 fold ↑ IFNγ: modest ↑ IL-1β + IFNγ: additional ↑ Aβ_1–40_: 2–12 fold ↑ Aβ_1–40_ + IFNγ: no- additional ↑ Aβ_1–42_: no effect α-MSH: ↑ EPA: ↑ HCV NS3: ↑	Sizable amount /(mRNA level)	HIV-vector: ↑ HIV-U937: ↑	[17, 20, 21, 30, 36, 37, 40, 43, 47, 48]
TNFα	ND	ND	4–8/ND	Aβ_1–40_: no effect Aβ_1–42_: modest ↑ HCV NS3: ↑	(mRNA level)		[17, 21, 37, 40, 43, 44, 47]
IL-1α			ND	Aβ_1–40_: no effect			[47]
IL-1β			ND	Aβ_1–40_: no effect HCV NS3: ↑	ND /(mRNA level)		[21, 30, 40, 47]
Caspase-1			ND	Aβ_1–40_: no effect			[47]
IL-12				LPS:↑			[52]
IFNγ				LPS:↑	ND		[30, 52]
iNOS			16% of cells (positive immunoreactivity)		(mRNA level)		[20, 36]
ROS					Sizable amount	HIV TAT-C protein: modest ↑	[20, 21]
Antinflammatory molecules							
IL-4					ND		[30]
IL-10			5,4	Aβ_1–42_: no effect	ND		[30, 43]
TGFβ1			~ 8		(mRNA level)		[20, 37]
TGFβ2			~ 100				[37]
Chemokines							
CCL2						HIV-U937: ↑	[30]
CCL5					Sizable amount	HIV-vector: ↑ HIV-U937: ↑	[30]
IL-8				HCV NS3: ↑			[40]
CXCL10						HIV-vector: ↑ HIV-U937: ↑	[30]

Levels of cytokines and chemokines assessed in the incubation media are reported. Data are expressed as pg/ml ± SD secreted in 24-h incubation experiments. *ND*, not-detectable. *↑*, increased production. Several inflammatory genes (i.e., iNOS, IL-1β, TNFα, IL-6, MHCII antigens, ARG1, and IL-10) were found to be expressed at the mRNA levels, in the CHME3 cells when co-cultured with differentiated neuronal SH-SY5Y wild type cells for 24, 48, 72 h. These markers were significantly upregulated in presence of the SH_swe_ differentiated neuronal cells, with major modifications observed for IL-1β and IL-6 gene expression at 72 h [50]. In addition, the mRNA levels of both pro-inflammatory (IL-1β, IL-6, and TNFα) and anti-inflammatory (IFNβ, IL-4, and IL-10) cytokines increased in response to CHME3 cell infection with the Japanese Encephalitis Virus (JEV) [53, 54]

### Cell morphology

A description of the morphology of the immortalized microglial cells was initially reported by Janabi and colleagues [17]. The authors did not distinguish among different clones, and in general they described the immortalized cells as “*globular or elongated with thick processes and numerous dark granulation in a large and clear cytoplasm*”, page 106. Cells were originally maintained in Eagle’s minimum essential medium (EMEM), containing 6 g/l glucose, 10% fetal calf serum (FCS), 2 mM glutamine, and antibiotics. When cultured on fibronectin-coated coverslips, the human microglial CHME3 cells showed mostly a globular morphology, detected by rhodamine-phalloidin staining, with a more intense signal surrounding the nucleus [35]. Interestingly, changes in the actin polymerization were induced by short term exposure (60 min) to human chemokines (MCP-1 or RANTES, 20 ng/ml), thus suggesting the ability of human microglia to migrate in response to chemotactic gradients. In contrast to the CHME3 cells, rat microglia displayed a more complex and branched morphology in response to rat chemokines, with an intense rhodamine-phalloidin staining in pseudopodia-like structures. However, both human and rodent microglia were able to migrate in response to several human chemokines showing a similar pattern, albeit the CHME3 cells responded to lower concentrations and in shorter times [35]. The CHME3 cells have been mostly maintained in Dulbecco’s modified MEM (DMEM), retaining their morphological features (i.e., globular cells with dark cytoplasmic vacuoles) [36, 37]. On the other hand, a phase-contrast image of the HMC3 cells, published later by Etemad and colleagues [29], showed the presence of different morphological phenotypes in the same culture, including globular, bipolar, and very elongated cells. Noteworthy, these cells were maintained in a more enriched medium (i.e., DMEM-F12) containing also a higher percentage of FCS (15% FCS).

### Antigenic profile

The antigenic profile of the human microglial clone 3 cells, as it emerges from published studies, is outlined in Table 1. In line with the original characterization [17], it was later confirmed that the CHME3 cell line were CD68 EBM/11 positive and GFAP negative [35]. Consistently, the expression of CD68 was detected in resting CHME3 cells using a different primary antibody (DAKO, UK) [38]. In addition, a small percentage (around 5%) of CHME3 cells was found to express the MCHII antigen under basal conditions [37], and resting CHME3 cells were described as MHCII and CD11b positive by flow-cytometry [39]. Expression of CD11b and lack of GFAP was further confirmed at transcriptional level [40]. In addition, it was shown that resting CHME3 cells expressed different toll-like receptors (TLRs), including TLR1, TLR2, and TLR 6; and that exposure to the HCV NS3 viral protein significantly increased the expression level of TLR2 and 6 [40]. Expression of the M-CSF receptor was detected in the CHME3 cells, by western immunoblot analysis [41]. Although the concept of microglial polarization has been recently challenged [42], the expression of specific M1- (like CD40 and CD86) and M2- (like CD163 and CD206) polarization markers has been detected in the CHME3 cells [37, 39, 43]. The majority of the CHME3 cells (approximately 30–60%) indeed express the CD40 antigen, whereas other markers (i.e., CD86; CD80; MHCII) were found in approximately 5–20% cells under basal conditions [37, 43]. Interestingly, differential expression of these markers was linked to different microglial activation states and functions. The stimulation of CHME3 cells with the amyloid beta peptide, Aβ_1–42_ (1 μg/ml, consisting mainly of monomers and dimers), reduced the expression of CD40, leaving largely unaffected the levels of other polarization markers [43]. Interestingly, microglial cells phagocytosing Aβ_1–42_ mostly expressed M2-polarization markers, i.e., CD163 and CD206 [43]. At higher concentrations (10–100 μg/ml), the Aβ_1–42_ tended to form larger oligomers, together with fibrillary aggregates [44]. In these conditions, the Aβ_1–42_ peptide (10 μg/ml) seemed to favor microglial M1-polarization, as demonstrated by increased expression of CD40 and CD11b, and no substantial effects on the expression levels of several M2-polarization markers, including CD163, CD206, CD200R (a deactivating receptor) and CD33 (an endocytic marker) [39]. When cultured in presence of neuronal precursor cells (NPCs), the CHME3 significantly upregulated the expression of MHCII antigens (up to 30%) together with the M2-polarization marker, CD206 [37]. Finally, treatments of CHME3 with conditioned-medium (CM) harvested from different glioma cell lines, i.e., U251, U87, LN229, U373, and A172 cells, resulted in the down-regulation of M1-specific (TNFα and CXCL10) and up-regulation of M2-specific polarization markers (IL-1 receptor antagonist and CD204) [41]. These authors further show that M-CSF released by glioma cells increased the pro-angiogenetic properties of microglial CHME3 cells with no significant effects on their polarization [41].

The research group of Prof. Krause (Department of Pathology and Immunology; Faculty of Medicine, University of Geneva), i.e., the depositor of the HMC3 (ATCC®CRL-3304) cell line, further characterized the antigenic profile of the HMC3 cells by immunofluorescence staining for different myeloid lineage markers and for resting/activated microglial receptors. The authors reported that “*resting HMC3 cells were strongly positive for the microglia/macrophage marker IBA1 (Fig.* *1a), positive for the endotoxin receptor CD14 (Fig.* *1b), but negative for the astrocyte marker GFAP (Fig. 1c and d). Markers of activated microglia, namely MHCII (Fig. 1e and f), CD68 (Fig. 1g and h) and CD11b (Fig. 1i and j), were negative in resting HMC3 cells, but upregulated after activation by IFNγ (10 ng/ml, 24 h)*”, page 575 [20]. Of note, the same description is reported in the data sheet of the HMC3 (ATCC®CRL-3304) cell line. In our opinion, data shown by Li and colleagues in figure 1 of their paper [20] seem to indicate a similar low level of expression of both IBA1 and CD14 in the HMC3 cells under resting conditions. Moreover, in contrast with the original characterization [17], resting HMC3 cells were found negative for the expression of CD68 and CD11b, albeit these activation markers were induced by IFNγ. Taken together, these data suggest that the HMC3 may have lost some of the original antigenic characteristics [17]. However, it should be noted that the expression level of CD68 in this culture appears to vary in function of the specific primary antibody adopted, as discussed above. Consistently, a basal expression of CD68 was observed in the HMC3 cells (provided by Prof. Krause) by flow cytometric analysis using a specific antibody produced by BD-Pharmingen, CA, USA [29]. In this study, it was also confirmed that resting HMC3 cells express IBA1, and at very low level other myeloid markers, including MHCII (HLA-DR), CD14, and CD45 [29]. The latter is a transmembrane glycoprotein expressed by cells of hematopoietic origin, except erythrocytes. It has frequently been used to distinguish CNS resident microglia (CD11b^+^/CD45^low^) from peripheral macrophages (CD11b^+^/CD45^high^) [45]. However, it has recently been shown that the expression level of CD45 on microglial cells may vary under pathological conditions, and novel specific microglial biomarkers have been identified [1, 2].

In addition, the cell line was characterized for the expression of several chemokine receptors. Particularly high levels of the CCR3, CXCR1, and CXCR3 receptors were expressed by the CHME3 cells, whereas the CCR2 receptor was barely detected [38]. Compared to primary human microglial cells, harvested from adult human brain tissue, the expression pattern of chemokine receptors in the CHME3 cells was very similar, with CCR3, CXCR1, and CXCR3 mainly distributed on the cell surface, and CCR5 only detectable in intracellular vesicles. Moreover, 24-h treatments with the pro-inflammatory cytokines, TNFα (25 ng/ml) or IFNγ (200 U/ml), induced a significant upregulation of CCR3 and a slight increase in the expression levels of CXCR3, suggesting a possible role of these receptors in CHME3 inflammatory activation [38]. These evaluations were performed by flow cytometry and, more importantly, all the antibodies used were specific for human antigens, i.e., no cross-reactivity with rodent antigens reported in their respective datasheets. This can be taken as an indirect evidence of the human origin of these cells, thus excluding hypothetical contaminations with rodent cells [19]. Analysis of the mRNA steady state levels largely confirmed cytometric observations and documented in addition a significant increase in the mRNA levels of CCR10 in response to TNFα. Though the CCR10 receptor protein could not be measured by flow cytometry (due to the lack of specific antibodies at that time), an indirect evidence of the expression of this receptor on the surface of the CHME3 cells was obtained by evaluating the migration capacity of the cells in response to IP-10 [38]. Noteworthy, it should be considered that in this study, the human primary microglial cells were maintained in medium containing GM-CSF (Leucomax, Norvartis), at final a concentration of 25 ng/ml. Moreover, it is stated that the CHME3 cells were grown in the same conditions as primary cultures. Thus, it is possible that the expression profile of chemokine receptors reflects the exposure to this relevant growth factor. Consistently, it has been shown that the HMC3 cells express at high levels the CXCR1, CXCR3, and CCR5 chemokine receptors, albeit also in this study, the cells were maintained in a highly enriched medium and at higher (15%) FCS concentrations [29]. In these conditions, the expression level of CCR2 was very low, whereas the HMC3 cells expressed at higher levels, other chemokines receptors, including the CX3CR1 [29]. The latter is currently regarded as a more specific microglial marker [2].

Finally, the expression of classic neurotransmitter receptors has been detected in resting CHME3 cells, with respect to the cholinergic receptor α7nAcR [44]. Interestingly, the expression level of this receptor varied in response to different inflammatory stimuli, including an up-regulation with LPS treatments and down-regulation in response to 10–100 μg/ml Aβ_1–42_. On the other hand, the observation of a direct inhibition of cFos expression obtained with dexmedetomidine, a specific α2 adrenergic receptor agonist, would suggest the presence of these receptors on the HMC3 cell membranes [32]. In addition, it has been documented the expression of functional neurotensin receptor 3 in the aliquot of cells named C13-NJ [46]. Accordingly, neurotensin stimulated microglial migration and cytoskeleton remodeling, via activation of ERK signaling.

### Functional properties

#### Cytokine and chemokine production

A summary of the inflammatory mediators produced by the cell line is provided in Table 2. In agreement with the original characterization [17], the CHME3 cells were found to release sizable amounts of IL-6 in the incubation medium, with a very low or undetectable secretion of other inflammatory molecules, including TNFα, IL-1α, IL-1β, and caspase-1 [47]. Consistently, the proportion of resting cells showing a positive immunoreactivity for IL-1β and its receptor, IL-1RI, was quantified as 2.9% and 12.5% of the total, respectively [36]. The production of TNFα was later estimated to be in the range of 4–28 pg/ml, 24 h after plating under basal conditions [43, 44]. On the other hand, basal IL-6 production by CHME3 cells was far more abundant and varied between 20 and 950 pg/ml in serum free medium after 24-h incubation [36, 43]. Similar results with respect to IL-6 and TNFα basal production were also reported by Liu J and collaborators [37]. Finally, it was detected under basal conditions, a marginal secretion of anti-inflammatory cytokines, including IL-10 (around 5.4 pg/ml) [43], TGFβ1, and TGFβ2 [37].

Interestingly, the CHME3 cells responded to different pro-inflammatory stimuli with significant increases in the production of IL-6. Two- to four-fold increases of basal production were measured in response to the bacterial endotoxin (LPS), used in the concentration range of 10–5000 ng/ml [48]. Similarly, the Aβ_1–40_ peptide, another peptide involved in the Alzheimer’s disease pathogenesis [49], induced a two-fold increase of basal IL-6 production at 10 μM and a 10–12-fold increase at 20–60 μM. The effects of Aβ_1–40_ were selective on the IL-6 pathway, and the secretion of other inflammatory mediators (i.e., TNFα, IL-1α, IL-1β, and caspase-1) was not modified [47]. However, while LPS did not affect cell viability, treatment with Aβ_1–40_ significantly reduced CHME3 viability, assessed by the MTT reduction assay [47, 48]. Consistently, the CHME3 microglial cells displayed a pro-inflammatory phenotype when co-cultured with human neuroblastoma cells SH-SY5Y stably expressing the amyloid beta precursor protein (APP) harboring the APP_695_ Swedish mutation (SH_swe_) [50]. Similarly to wild type cells, the SH_swe_ cells can be differentiated into neurons by exposure to 10 μM retinoic acid for 7 days. However, the SH_swe_ neurons produce increased amounts of immature APP, soluble APP, and the Aβ_1–40_ peptide than wild type cells, and this can explain their pro-inflammatory effects when co-cultured with CHME3 cells. Briefly, the CHME3 cells were found to express, at the mRNA levels, several inflammatory genes (i.e., iNOS, IL-1β, TNFα, IL-6, MHCII antigens, ARG1, and IL-10) when co-cultured with differentiated neuronal SH-SY5Y wild type cells for 24, 48, and 72 h. These markers were significantly upregulated in presence of the SH_swe_ differentiated neuronal cells, with major modifications observed for IL-1β and IL-6 gene expression at 72 h [50]. Interestingly, the expression of several microRNAs (miRNAs) implicated in microglial polarization (i.e., miR-155, miR-146a, and miR-124), together with their respective downstream signaling pathway, was modulated in the CHME3 cells co-cultured with SH_swe_ neuronal cells [50]. Conversely, the Aβ_1–42_ peptide, at concentrations lower than 5 μg/ml, did not exert any stimulatory effect on IL-6 production [36, 43], neither modified basal TNFα and IL-10 secretion by the CHME3 cells [43]. Only a modest increase of basal TNFα production (from 4 pg/ml to 6 pg/ml) was observed in response to higher concentrations (10 μg/ml) of the Aβ_1–42_ peptide [44]. With respect to IL-6 production, IL-1β seems to be the most effective inducer, increasing by 50 times the basal release (when used at 50 ng/ml, for 24 h) [36]. On the other hand, IFNγ per se displayed only modest stimulatory effects [36, 47]. In contrast to what was observed in rodent models [51], IFNγ did not modify the response of the CHME3 cells to 10 μM Aβ_1–40_ peptide [47], whereas it significantly increased the pro-inflammatory activity of IL-1β (both cytokines used in combination at 50 ng/ml) [36]. Neither IL-1β nor IFNγ per se affected microglia viability (MTT assay) [36, 47], albeit the cytokines used in combination significantly reduced cell viability [36]. Finally, a modest stimulatory effect on IL-6 production was observed in response to the α-melanocyte stimulating hormone (αMSH), and this was potentiated by a specific receptor agonist [47]. The ω-3 fatty acid, eicosapentenoic acid (EPA), displayed stimulatory effects only at lower concentrations (5–10 nM), an effect that was reversed by cell exposure to Aβ_1–42_ peptide [43]. Another relevant ω-3 fatty acid, docosahexaenoic acid (DHA), did not show any significant stimulatory effect on IL-6 production [43]. In addition, the CHME3 cells were found to release other cytokines, including IL-12 and IFNγ, in response to LPS [52].

A potent inducer of CHME3 cell activation is the non-structural NS3 protein of the hepatitis virus C (HCV). The recombinant HCV NS3 (at 2–20 ng/ml) promoted the upregulation and significantly increased the release of several cytokines, i.e., IL-1β, IL-6, and TNFα, and the chemokine IL-8 [40]. The effects of NS3 protein were mediated by binding to TLRs, as TLR2 and TLR6, followed by activation of downstream NFκB signaling pathway. TLR specific agonists inhibited the pro-inflammatory effect of the HCV NS3 protein by promoting the release of anti-inflammatory IL-10 [40]. Similarly, the mRNA levels of several cytokines, with both pro-inflammatory (IL-1β, IL-6, and TNFα) and anti-inflammatory (IL-4 and IL-10) activities, increased in response to microglial infection with the Japanese encephalitis virus (JEV) [53, 54], together with a significant up-regulation of type I interferon, IFNβ. Increased levels of IFNβ mRNA were mediated by activation of the transcription factor IRF3 [55] and subsequent expression of IRF8 [53]. JEV-mediated microglial infection is also characterized by the induction of TRIM21, which exerts an inhibitory role on microglial antiviral responses [55]. In addition, a more comprehensive characterization of the dynamic changes occurring in the human microglial microRNAome and transcriptome in response to JEV infection can be found in the paper by Kumari and collaborators [54]. This study further extends previous observations, confirming the immune regulatory role of different microRNAs, i.e., miR-155 [53], miR-146a [56], and miRNA-432 [57]. Consistently, microarray data analysis showed the downregulation of STAT1 gene during JEV infection, which suggests impaired signaling downstream of cytokine receptors/JAK activation [58]. Thus, a complex interplay between miRNAs, transcription factors and mRNAs occur during JEV infection, contributing to the inhibition of microglial antiviral responses (as for example secretion of type I interferon) and facilitating viral replication in CHME3 cells.

The expression of different inflammatory molecules, including IL-1β, IL-6, TNFα, iNOS, vascular endothelial growth factor (VEGF), and TGFβ1, was documented, albeit only at the mRNA level, in the HMC3 cells [20, 21]. The expression levels of IL-6 were reduced by silencing the isoform 4 of NADPH oxidase (NOX4), thus, suggesting that the endogenous production of free oxygen radicals is linked to basal expression of IL-6 [20]. The basal mRNA levels of other genes, i.e., iNOS, VEGF, and TGFβ1, was unaffected by downregulation of NOX4. Consistently with data obtained using the CHME3 cells, the mRNA steady state level of the pro-inflammatory cytokines (IL-1β, IL-6, and TNFα) was modulated by a specific microRNA (miR-17, as discussed in more detail in the next section) [21]. In addition, it has been shown that the HMC3 cells (provided by Dr. J Pocock, University College of London) produce significant amounts of IL-6, together with several chemokines (i.e., CXCL10, CCL5, and CCL2) in response to human immunodeficiency virus (HIV) vector-transduced monocytoid cells (HIV-U937 cells). HMC3 microglial cells in contact with HIV vector alone responded with increased production of IL-6, CXCL10, and CCL5, albeit to a lower extent in comparison to activation with HIV-U937 cells [30]. Data presented in this paper confirm a basal production of IL-6 in HMC3 cells, together with sizable amounts of CCL5. Other cytokines, including IFNγ, IL-1β, IL-4, and IL-10 were not detected [30]. The immunomodulating agent, teriflunomide, significantly reduced microglial inflammatory activation induced by HIV-U937 cells, whereas, monomethylfumarate (MMF) was less effective (only CXCL10 reduction at the highest dose tested). Both drugs were also able to inhibit cytokine release by microglial cells activated in response to the HIV-vector, with MMF displaying a more relevant effect. Interestingly, data obtained with the HMC3 cells were confirmed in primary cultures of human microglial cells (isolated from patients with intractable epilepsy), thus further supporting the relevance of this experimental model to study human microglial properties. Conditioned medium harvested from HMC3 cells exposed to HIV transduced monocytoid cells caused significant neuronal toxicity, whereas neuroprotection was observed with teriflunomide and MMF treatments [30]. However, Rawat and collaborators [59] questioned the use of human microglial cell lines, including the HMC3 cells, for the study of HIV pathogenesis due to the genetic manipulation caused by the immortalization procedure, the higher growth rate in comparison to primary microglial cells, and reduced HIV replication within these cells. These authors further characterized the human primary microglial cell model originally developed by Etemad and colleagues [29], thus deriving human microglial cell cultures by peripheral monocytes exposed to a cocktail of human recombinant cytokines (i.e., M-CSF, GM-CSF, NGFβ, and CCL2). These cells were efficiently infected by HIV, and more importantly, supported long-term HIV infection promoting the release of infectious virions into culture media [59]. However, immortalized microglial cells were recently used to study HIV latency and its regulation. In this study, adult human microglial cells were immortalized using a combination of SV40 and hTERT; the latter reduced the rate of cell growth and basal microglial activation. Latently infected microglial cells displayed marginal expression of HIV, but can be promptly activated in response to different inflammatory stimuli (TNFα, IL-1β, and LPS) [19] On the other hand, CHME3 cells were used to test the translational efficiency of highly conserved elements on the HCV viral genome, i.e., the internal ribosome entry site (IRES) [60]. In this study, it was demonstrated that the ability of HCV to replicate in microglial cells, together with the property of IRES sequences to change in order to favor viral latency in the CNS and full replication in the liver. At variance of murine BV2 microglial cells, the CHME3 displayed increased resistance to the cytotoxic effects of 5Z-7-oxozeaenol, an inhibitor of TGFβ-activated kinase 1 [61]. Finally, the CHME3 cells were used to identify molecular factors involved in Zika virus cell entry [62].

#### Free oxygen and nitrogen species production

Rodent microglial cells have been extensively characterized for their ability to produce reactive oxygen and nitrogen species, including NO mainly generated in the μM range by the prompt upregulation of iNOS in response to different pro-inflammatory stimuli. In contrast, only a small proportion (around 16% of the total) of CHME3 cells was found to express iNOS 24 h after the plating under basal conditions (Table 2). Moreover, the total fraction of iNOS positive cells did not vary in response to the Aβ_1–42_ peptide, even though a larger percentage of iNOS^+^/Aβ_1–42_^+^ cells was detected at different time points (24-96 h incubations) [36]. These data would therefore suggest that the expression of iNOS in the CHME3 cells is related to their phagocytic capacity [36]. Detection of iNOS mRNA was shown in CHME3 cells co-cultured with differentiated SH-SY5Y neuronal cells [50], as well as in resting HMC3 [20] (as discussed above). However, NO production was not evaluated in these studies, neither in other published articles. Thus, there are no available evidence that the CHME3/HMC3 cells release NO under basal conditions or in response to inflammatory activators. On the other hand, it has been shown that the HMC3 cells spontaneously release sizable amount of free oxygen radicals (ROS) ([20]; Table 2). Noteworthy, cells were maintained according to the original protocol [17], in EMEM supplemented with 10% FCS, and antibiotics, at 37 °C in a 10% CO_2_ humidified atmosphere. The production of ROS appeared to be mediated by NOX4 activity, and it was not significantly modulated by short term (2 h) treatments with IFNγ [20]. Consistently, Jadhav and collaborators have shown a basal production of ROS by the HMC3 cells, with a modest (0.15-fold) increase induced by treatment with the HIV TAT-C protein ([21]; Table 2). Specifically, the viral protein was also able to reduce the expression of miR-17 in the HMC3 cells, an effect that paralleled with increased expression of the NOX2 and NOX4 enzymes. In fact, the 3′ untranslated regions (UTRs) of both genes contain multiple binding sites for miR-17. Thus, miR-17 overexpression in HMC3 cells significantly reduced the expression of NOX2 and NOX4 proteins, whereas the transfection of anti-miR17 oligonucleotides displayed opposite effects on both enzyme expression and activity. Similar effects were also observed with respect to the expression of certain pro-inflammatory cytokines, i.e., IL-1β, IL-6, and TNFα [21]. Interestingly, this research group had previously shown the ability of the HIV TAT-C protein to modulate the expression of miR-32 and TRAF3 in human microglial cells [63]. However, in this previous study, the authors employed the CHME3 cells (no source is listed in the paper), whereas later used the HMC3 cells provided by Prof. Krause [21]. Notably, in both cell lines, the HIV TAT-C protein had similar pro-inflammatory effects, by promoting microglial activation through regulation of micro RNA expression.

#### Phagocytosis

As discussed above, the HMC3 cells displayed lower phagocytosis activity in comparison to the primary culture used for the immortalization procedure [17]. The phagocytic ability of the CHME3 cells was later confirmed by exposing the culture to fluorescent Aβ_1–42_ (at concentrations < 5 μg/ml) [36, 39, 43]. In a first study, it has been shown that the CHME3 cells were able to uptake Aβ_1–42_ aggregates. The activity was particularly relevant at 5 μg/ml concentrations of Aβ_1–42_, despite a significant increase in cell toxicity [36]. As discussed above, phagocytic CHME3 cells displayed mostly M2-polarization markers, including CD163 and CD206 [43]. Pre-incubation with IFNγ alone or in combination with IL-1β, further increased the Aβ_1–42_ phagocytic capability of the CHME3 cells. In contrast, IL-1β per se had no effects on the CHME3 phagocytic activity, despite its relevant stimulatory action on IL-6 production (as discussed above) [36]. Taken together, these data suggest that IFNγ and IL-1β may selectively modulate certain microglial functions, whereas in combination can synergistically act to promote both cytokine secretion and phagocytosis. Interestingly, the Aβ_1–42_ phagocytic ability of the CHME3 cells was significantly increased by treatment with the ω-3 fatty acids, DHA, and EPA [43], and by the pro-resolving inflammatory lipid mediator, maresin (MaR1), a derivative of DHA, in short-term incubation (1–6 h) experiments [39]. In the CHME3 cells, phagocytosis was also assessed by exposure to latex beads and subsequent quantification by flow cytometry and confocal microscopy [37]. The phagocytic ability of microglial cells significantly increased when cells were cultured in presence of human NPCs [37]. Interestingly, it has been recently shown that the CHME3 cells can reduce the extent of extracellular accumulation of sAPPα and Aβ_1–40_ when co-cultured with differentiated SH_swe_ neuronal cells [50]. However, the ability of microglial cells to clear these neuronal products appeared to be saturated after 24 h, with accumulation observed after longer incubations (48–72 h). In co-culture with SH_swe_ neurons, CHME3 microglial cells progressively up-regulated senescence markers, an effect that can possibly explain the reduced efficiency in the Aβ clearance [50]. Noteworthy, the CHME3 microglial cells were also able to efficiently internalize exosomes released from differentiated neuronal cells. In this regard, exosomes derived from SH_swe_ neurons were characterized for a more electron dense content in comparison to exosomes released by wild type differentiated SH-SY5Y cells, and more importantly for their enrichment in miRNAs involved in inflammatory processes. Interestingly, when transferred to microglial cells, neuronal exosomes tended to co-localize with lysosomes. However, reduced lysosomal co-localization was observed when microglia were treated with SH_swe_-exosomes in comparison to their wild type counterpart, thus suggesting dysfunctional lysosomal activity in microglial cells. Reduced lysosomal function can affect the rate of protein degradation by microglia, thus favoring Aβ accumulation. In addition, exosomes derived from SH_swe_ cells were more toxic than those released by SH wild type cells, and microglial cells appeared to be activated in response to SH_swe_-exosomes [50]. Finally, it has been shown that the HIV TAT-C protein is efficiently internalized by the CHME3 cells, and can be detected within cell nuclei [63].

Consistently, the HMC3 cells were also found to have phagocytic properties [29]. In this study, the HMC3 cells were exposed to tetramethylrhodamine (TRITC)-labeled *Staphylococcus aures*, and phagocytosis was assessed by flow cytometry and confocal microscopy. The HMC3 displayed a significant capacity to uptake the bacterial particles, with two distinct populations indeed detected by flow cytometry. However, the overall phagocytic activity of HMC3 cells was lower if compared to peripheral dendritic cells [29].

#### Migratory capacity and secretion of matrix metalloproteases

It is currently known that chemokines play an important role in the regulation of myeloid cell function, particularly in the control of cell migration within tissues during inflammation, but also in development and tissue repair [64]. In particular, chemokines can regulate macrophage/microglia migration to the site of inflammation and in the CNS; they mediate the crosstalk with different cell types, for example, between microglia and neurons or astrocytes [65]. Accordingly, the expression of specific chemokine receptors was observed in both the CHME3 and the HMC3 cells, with a similar pattern [29, 38]. Consistently, the CHME3 cell were able to migrate in response to different chemokines (i.e., MCP-1, MIP-1α, MIP-1β, RANTES, IL-8, and IP-10) [35], albeit IP-10 seemed to reduce cell viability, measured by MTT assay [38]. Furthermore, matrix metalloproteases (MMPs) are important enzymes that favor the remodeling of the extracellular milieu, thus increasing immune cell motility and migratory capability [66]. Consistently, it was shown that the CHME3 cells can secrete in the incubation medium sizable amounts of MMP2 under basal conditions [67]. The release of MMP2 could be augmented by treatment with different human chemokines, including MCP-1, MIP-1β, RANTES, and fractalkine (albeit to a lower extent) or in response to IL-1β (1–50 ng/ml). Chemokines were also able to increase the release of specific tissue MMP inhibitors, namely TIMP1 and 2, by the CHME3 cells [67]. A modest release of MMP9 was detected by zymography (since specific ELISA was unavailable, at that time). In addition, MMP activity (measured by zymography) was further increased by different chemokines, particularly MCP-1 and IL-8, and by treatment with TNFα (1–10 ng/ml). This study, however, showed a certain degree of variability in the CHME3 responses, with functional effects of some chemokines observed in some experiments but not in all [67]. Moreover, the CHME3 cells released increased amounts of MMP-1 and MMP-3 in response to CM, harvested from human peripheral blood monocytes infected with *Mycobacterium tuberculosis* (coMTb), in a concentration and time-dependent manner [68]. The stimulatory effect of coMTb was mediated by activation of p38 MAPK, but possibly involved other kinase signaling pathways, including ERK and AKT. Interestingly, basal ERK activity was detected in CHME3 cells [68]. In addition, the CHME3 cells express ADAMTS-13, an enzyme belonging to the ADAMTS family of metalloproteases and mainly synthesized in the liver [69]. The expression level of ADAMTS-13 in the CHME3 cells was lower in comparison to other human brain cell lines, including the neuroblastoma SH-SY5Y cells, the glioblastoma U373 cells, and the human brain endothelial cells, hCMEM/D3. Moreover, the levels of ADAMTS-13 were significantly reduced by treatments with IL-1β in the U373 cell line, whereas a trend towards reduction was also observed in the CHME3 cells. Other cytokines, including IL-6 and TNFα, had no inhibitory effects [69].

#### Other properties

The CHME3 cells express different isoform of lipooxygenase (LOX) enzymes, including the 15-LOX-2 and the 5-LOX. Accordingly, a basal production of pro-resolving inflammatory mediators have been observed, including lipoxin A4 (LXA4, approximately 250 pg/ml) and resolving D1 (RvD1, approximately 200 pg/ml) [44]. In addition, the CHME3 cells have been also characterized for their ability to produce neurotrophic and growth factors. A basal release of brain derived neurotrophic factor (BDNF) was assessed and quantified within the range of 8–300 pg/ml 24 h after plating [36]. Similar results on basal BDNF production were also later shown [37]. Interestingly, the Aβ_1–42_ peptide (5 μg/ml) significantly reduced the release of BDNF by CHME3 cells, an effect that can potentially exacerbate neuronal damage [36]. However, when tested at lower concentrations, the Aβ_1–42_ peptide did not modify the secretion of BDNF [36, 43]. Interestingly, the release of BDNF was augmented by treatment with the ω-3 fatty acid EPA, whereas DHA had no significant stimulatory effect [43]. CHME3 cells treated with glioma CM were able to release the VEGF-A together with the insulin-like growth factor binding-protein 1. Both factors were implicated in the pro-angiogenetic effects of microglial cells [41].

The cell line was characterized for the ability to influence the proliferation of other cells, including NPCs, endothelial cells, and lymphocytes. An important cross-talk between microglial CHME3 cells and NPCs, derived from 5 to 7.5 weeks old human embryonic CNS tissues, was found using in vitro co-cultures [37]. In this experimental paradigm, microglial CHME3 cells significantly increased the metabolic activity of NPCs and reduced cell death. In addition, the CHME3 cells significantly sustained NPC proliferation, inhibiting their differentiation towards the neuronal and astrocyte lineage. This effect was indicated by a lower percentage of PSA-NCAM^+^ (polysialylated neural cell adhesion molecule) and tubulin III^+^ cells on one hand, and lower counts of positive cells for the glial precursor markers, A2B5 and GFAP, on the other hand. Accordingly, the percentage of nestin positive cells, indicating the presence of undifferentiated NPCs, increased in presence of microglial CHME3 cells added to NPCs during the differentiation protocol. It was speculated that the inhibitory effect of CHME3 cells on NPC differentiation was mediated by the abundant secretion of IL-6 in the incubation medium [37]. Interestingly, when co-cultured with CHME3 cells, NPCs increased the expression of CD200, whereas the microglial cells upregulated the synthesis of its specific receptor. On the other hand, NPCs significantly increased the rate of microglial proliferation and the production of TGFβ, with a modest increase of IL-6 and a reduction of BDNF release by CHME3 cells [37]. Despite these data on IL-6 and BDNF, in general, pro-survival and anti-inflammatory effects were observed in the interaction between NPCs and microglial cells in vitro, supporting a possible neuroprotective role of human NPC-based therapies. The medium collected by CHME3 cells exposed to glioma U87 CM increased the ability of tube formation of human umbilical vein endothelial cells, and this pro-angiogenetic effect was more evident in comparison to the supernatant of resting CHME3, i.e., not exposed to glioma CM [41]. In addition, a modest, albeit not significant, stimulatory effect on lymphocyte proliferation was observed in vitro using the HMC3 cells [29]. Finally, the electrophysiological properties of the human microglial clone 3 cells have been described by Nicholson and Randall [70]. In this research paper the cell line is indicated under the acronym of C13-NJ. Cells showed a mean membrane capacitance of 20.7 ± 0.8 pF, and a mean resting potential − 49 ± 4 mV. In addition, the cells exhibited depolarization-induced inward currents, compatible with the expression of Na_v_ 1.5 sodium channel. However, no significant changes in these sodium current properties were observed in response to 12-h treatments with LPS (1 μg/ml) and Aβ_1–42_ (10 μM). Interestingly, these data are in contrast with evidence obtained using rat microglial cultures, further suggesting the importance of human microglial experimental models.

## The human microglial CHME-5 cell line

**Fig. 2:**
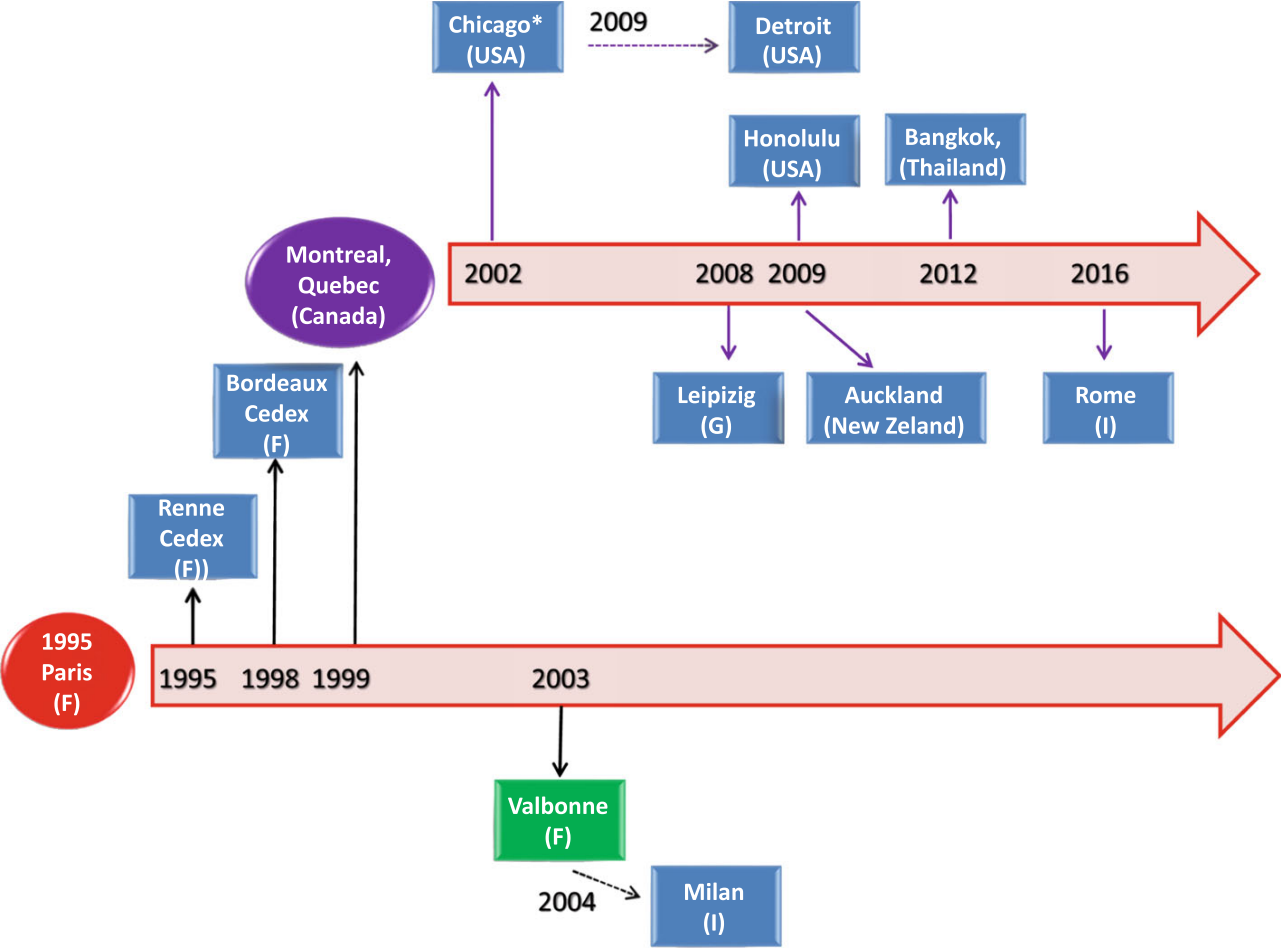
Historical reconstruction of the distribution process of the human microglial CHME-5 cell line. The human microglial CHME-5 cell line was developed in the laboratory of Prof. M Tardieu, Paris, in 1995 (red circle). As shown in the picture, CHME-5 cells have been distributed worldwide (blue boxes). Same cells were used with the acronym C13-NJ cells (green box). Distribution followed two main pathways, either directly from Prof. Tardieu’s laboratory (black thick arrows) or indirectly by the first recipient laboratory (black dotted arrows). Since 2002, the laboratory of Prof. Talbot, University of Quebec, Montreal, Canada, (purple circle), appears to be the main distributor of the CHME-5 cells. In addition, we identified several studies (not reported in the schematic), in which the CHME-5 cells were used without any indication of the source. Moreover, other laboratories received the cells from academic institutions for which publication records are unavailable.* Prof. Feinstein, University of Illinois, Chicago, USA, personal communication

The HMC3 cell line has been widely used under the denomination of human microglial CHME-5 cell line. A historical reconstruction of the distribution process of the CHME-5 cell line across different laboratories, based on published articles, is presented in Fig. 2. From this schematic, it appears that the cell line was originally distributed under the name of CHME-5 and used in several publications between the years 1995 and 1999. Later on, the CHME-5 cells have been mainly distributed by the laboratory of Prof. Talbot, University of Quebec, Montreal, Canada. Interestingly, the cell line was used in one publication under the name of C13-NJ [46] and distributed under the name of CHME-5 cells [71]. A number of publications have not cited the source of their cell samples whilst others received the cells from academic institutions for which publication records are not available. This may suggest that the cell line was distributed wider than it may be deducted from published articles.

The CHME-5 cells displayed same morphological [72, 73] and immunological properties [74], i.e., abundant production of IL-6 under basal conditions. These early reports showed the ability of CHME-5 cells to perform phagocytosis of latex beads, yeast, and zymosan particles [72, 74]. Moreover, the CHME-5 cells were characterized for a high rate of oxygen consumption [73, 75] and excessive production of ROS [76] under basal conditions. The CHME-5 cells were resistant to heat shock stress, via increased expression of heat shock proteins (Hsc70 and Hsp70), changes in their metabolism, mitochondrial function, and cytoskeleton remodeling [72, 77]. Accordingly, co-culturing of CHME-5 cells with rat C6 glioma cells promoted the relocation of the heat shock protein Hsp60 to the nucleus together with transient changes in microglial metabolism, including reduction of oxygen consumption, decreased mitochondrial enzymatic activities and membrane potential, increased lactate release, and reduction of ATP content [73]. In addition, the C6 glioma cells promoted microglial phagocytic activity and induced cytoskeleton modifications. Functional TREM2 receptors were detected in the CHME-5 cells [71]. Moreover, the CHME-5 cells expressed functional receptors for advanced glycation end-products (AGEs), named RAGEs [78]. AGEs increased ROS production in CHME-5 cells and reduced cell viability [79]. Consistently, exposure of CHME-5 cells to AGEs triggered microglial activation, as suggested by increased expression of the glucose transporter GLUT-5, induction of MHCII antigens, and increased TNFα release [78]. The CHME-5 cells were resistant to low-energy electromagnetic field exposure and did not show any particular changes [80]. In contrast, increased ROS production, increased expression of GLUT-5 and MHCII antigens, together with a reduction in cell viability were observed in response to high dose ionizing radiations [81].

It was shown that the CHME-5 cells were able to sustain the replication of the M-tropic HIV-1 strain Yu-2 with a similar efficiency to human peripheral blood monocytes [82]. In addition, HIV-1 infected CHME-5 cells displayed extended survival and were resistant to cell death induced by LPS in combination with the protein synthesis blocker, cycloheximide. The cytoprotective effect induced by HIV infection was mediated by the HIV TAT protein and subsequent activation of survival signaling depending on the AKT pathway [82]. Since this initial observation, the human microglial CHME-5 cell line has been widely used to study the biology of HIV-1, as documented by more than 15 publications [83–98]. The CHME-5 cells were resistant to murine norovirus MV1 infection [99], whereas they were successfully used to culture *Chlamydia pneumoniae* isolated from post-mortem brain tissue of two patients affected by Alzheimer’s disease [100]. The CHME-5 cells were susceptible to infection by human chikungunya virus [101], coronavirus (huCV) OC43 subgroup, and resistant to huCV-229E subgroup [102, 103]. In addition the CHME-5 cells could be infected by Zika virus that interferes with the mitotic fuse [104]. Interestingly, the CHME-5 cells were not able to sustain persistent huCV-OC43 infection, and responded to the acute infection with increased MMP activity and NO production [105]. In contrast to the HMC3/CHME3 aliquots, increased iNOS expression and NO production was detected also after overexpression of the endogenous retrovirus W envelope protein [88] and in response to a mixture of pro-inflammatory cytokines, including TNFα, IL-1β, and IFNγ [106]. Furthermore, the CHME-5 cells displayed increased arginase (ARG) activity in response to IL-4, which was significantly reduced by several antiretroviral drugs [106].

In contrast to all the evidence reviewed above, none of the following transcripts was detected in the CHME-5 cells by semi-quantitative RT-PCR using human specific primers: IL-1α and β, IL-6, IL-10, IL-12, TNFα, TGFβ, IFNγ, MIP-1α, and MCP-1 [105]. In addition, it has recently been shown by genotyping and amplification of a key human gene CyCT1 that several aliquots of CHME-5 cells (in use in different laboratories) are cross-contaminated by rat glioblastoma cells [19]. This observation further highlights the importance of authenticated cell lines as experimental models. This process, which has recently been performed by ATCC® on the HMC3 cells, ensures for the origin of the cell line excluding cross contamination by other species. In addition, it allows researchers to control the genetic drift that can possibly occur with serial passaging of the cells, contributing to cell heterogeneity in cultures [107]. Genetic drift may explain the subtle differences observed among different laboratories using the same cell line.

## The HMC3 (ATCC®CRL-3304) cell line

**Fig. 3:**
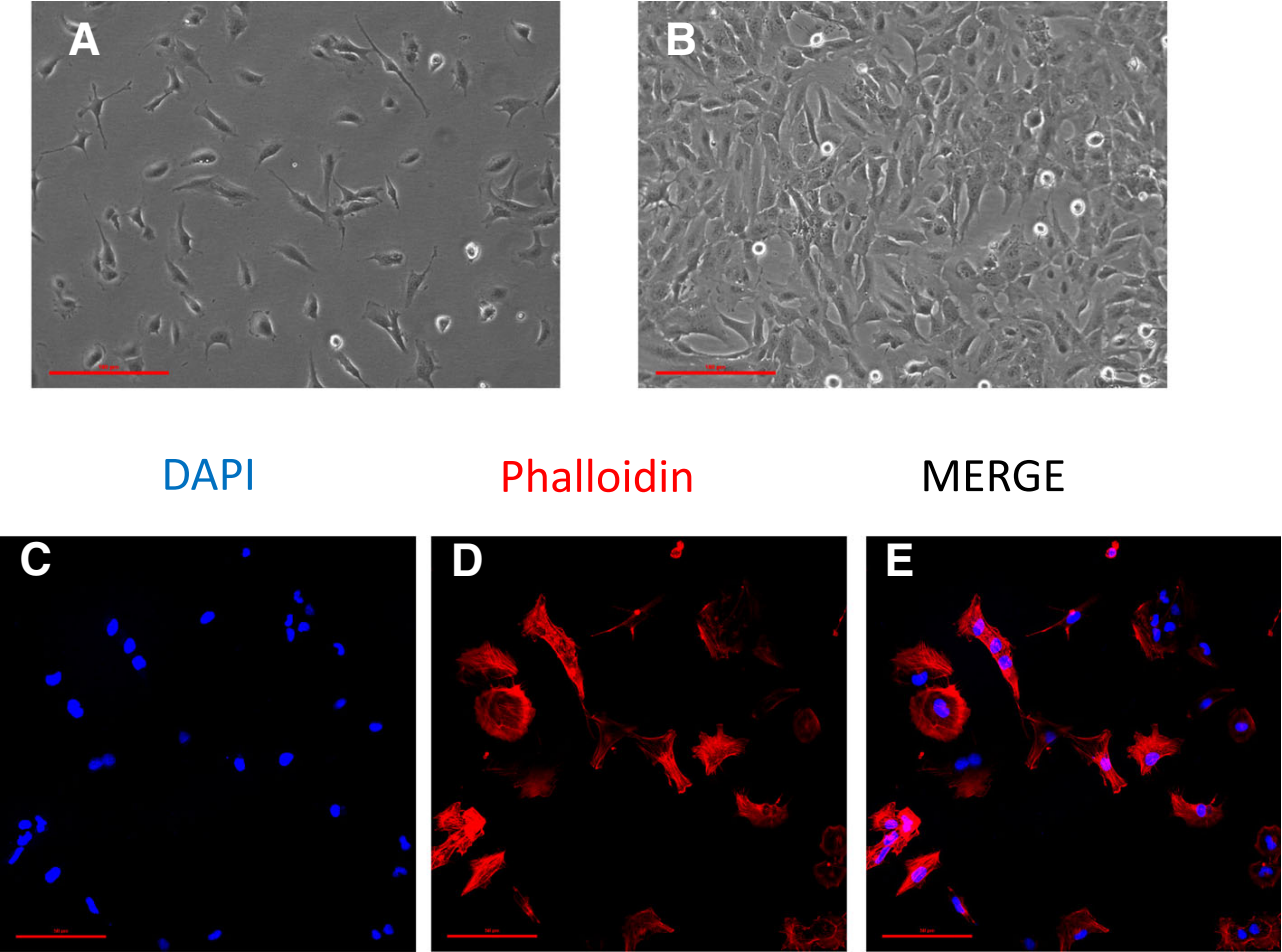
HMC3 cell morphology and labeling of cytoskeletal F-actin filaments. **a**–**b** The human microglial cell line HMC3 as it was observed by phase-contrast microscopy at in vitro day 1 (**a**), and when cells reached the confluency (**b**). × 10 magnification, scale bar 100 μM. **c**–**e** A representative example of confocal images (1024 × 1024 pixels) acquired at × 20 magnification with a confocal laser scanning system (A1+, Nikon). Cells were grown on glass coverslips for 24 h, and their morphology was evaluated by labeling the cytoskeletal F-actin filaments with tetramethylrhodamine (TRITC)-conjugated phalloidin (red fluorescence). Cells were counterstained with the nuclear probe, 4′,6-damidino-2-phenylindole dihydrochloride (DAPI, blue fluorescence). The merged image is shown in (**e**). Scale bar 50 μM

The human microglial cell line, HMC3 (ATCC®CRL-3304) (Lot/batch number 70002138), was delivered to our laboratory on June 7, 2017. A certificate of analysis was provided by ATCC® for the specific lot of cells delivered, which certifies the human origin of the cells and the absence of cross contaminating cells from other species. The latter was confirmed by the cytochrome C oxidase I interspecies assay (COI analysis), a PCR based method to detect species specific variants of the COI gene and to rule out inter-species contamination. In addition, the certificate provides a human unique DNA profile, by short tandem repeats analysis, which can be useful to control the cell line for possible phenomena of genetic drift (see above). The HMC3 (ATCC®CRL-3304) cells were immediately placed in liquid nitrogen as suggested by the distributor and 4 days later thawed following the instructions found in the data sheet. Additional information about the culturing conditions was obtained directly by the technical support of ATCC®. Cell culture reagents [Eagle’s Minimum Essential Medium (EMEM, ATCC® 30–2003™) and Fetal Bovine Serum (FBS, ATCC® 30–2020™)] were purchased from ATCC®, and cells were cultured in EMEM supplemented with 10% FBS and antibiotics (100 IU/ml of penicillin and 100 μg/ml of streptomycin, Biochrom AG, Berlin, Germany). The entire aliquot of frozen cells was plated in one Corning® T75 flask (Catalog #430641) in 15 ml complete growth medium (previously equilibrated for 2 h in the incubator) and incubated in a humidified atmosphere (5% CO_2_) at 37 °C. This aliquot was numbered using an internal code as passage 1. However, during this first passage in culture, we had to replace the growth medium more frequently than recommended to prevent excessive acidification. The pH of the incubation medium was measured at any medium change. Averaged pH value was 6.88 ± 0.02 (mean ± SEM, *n* = 13) after 24–48 h of incubation, regardless the number of cells. By avoiding the excessive acidification of the medium, cells remained viable and reached confluency within the times indicated by the ATCC®’s protocol. The technical support of ATCC® confirmed the tendency towards a quick acidification of the EMEM and more importantly reassured us on the healthy morphology of our cultures (Fig. 3). Cells were sub-cultured twice in order to have a homogenous population for the experiments and stored in frozen aliquots at passage 3 (p3). Mycoplasmas and the related Acholeplasmas (both referred as “mollicutes”) represent a frequent source of cell line contamination. These bacteria pass through standard 0.22 μM filter systems, their growth is not controlled by commonly used antibiotics and is not detectable under contrast phase microscopy. Thus, mollicutes can grow until extremely high titers without producing any turbidity in the supernatants. In addition, mycoplasma contaminations can significantly affect the biology of the cell culture, thus the quality of the experimental results [108]. Before freezing the cells, we excluded possible mycoplasma contaminations using the Venor®GeM Classic Mycoplasma detection kit, thus searching mycoplasma DNA in the incubation medium of almost confluent cultures at p3.

Based on our preliminary observations, we recommend to maintain the ATCC®CRL-3304 cell line as follows: cells should be plated at the density of 20,000 cells/cm^2^ in T75 flasks and kept in EMEM 10% FBS and antibiotics (complete growth medium, 15 ml/flask) by changing the medium the day after plating and every 24–48 h. Cells should be passaged twice a week, when reaching 90–95% confluency. Of note, the HMC3 cells can grow also in different media, including MEM (Biochrom, Germany) and in DMEM-F12 (Biochrom), with the addition of 10% FCS (Biochrom) and antibiotics. These media showed a stable pH in the neutral range, thus were replaced every 2–3 days. Both media contain indeed higher concentrations of NaHCO_3_, i.e., 2.2–2.4 mg/ml *vs* 1.5 mg/ml dissolved in the ATCC®EMEM medium. MEM was complemented also with 1 mM sodium pyruvate and 1X non-essential amino acids (Biochrom). When maintained in these media, cells divided more rapidly retaining their morphological features (not shown). For the phenotypical analysis, we plated the cells at 30,000 cells/cm^2^ in T25 flasks and let them grow for 3 days until almost confluent. IFNγ (10 ng/ml), when used, was added in the last 36 h. The expression of specific lineage markers was tested by immunocytochemistry and flow cytometry. The primary antibodies used for these evaluations are reported in Table 3. The expression of inflammatory genes was analyzed at the mRNA level by reverse transcription followed by real-time (Q)-PCR analysis, performed according to our standard protocol [109]. In brief, total cytoplasmic RNA was prepared from cells using TRIZOL reagent (Invitrogen) and 1 μg aliquots were converted to cDNA using random hexamer primers and the ImProm-II Reverse Transcriptase kit (Promega). Q-PCRs were performed using the following cycling conditions: 35 cycles of denaturation at 95 °C for 20 s; annealing at 60 °C for 30 s; and extension at 72 °C for 30 s; the Brilliant SYBR Green QPCR Master Mix 2X (Agilent); and the specific primers reported in Table 4. All the primer sets, except the one used for iNOS and COX2 expression, aligned only human sequences. Q-PCR reactions were carried out in a 20 μl reaction volume in a MX3000P real time PCR machine (Stratagene). Relative mRNA concentrations were calculated from the take-off point of reactions (threshold cycle, Ct) using the comparative quantitation method [109]. Ct values for β-actin (ACTB) expression served as a normalizing signal. ACTB was selected considering that it is a constitutively highly expressed gene in human microglia [2]. Q-PCR efficiency ranged between 94 and 106%. At the end of Q-PCR, the products were separated by electrophoresis through 2% agarose gels containing 0.1 μg/ml ethidium bromide to ensure the product was the correct size. For functional experiments, cells were plated at 30,000 cells/cm^2^ in 96 well plates (100 μl/well), let recover overnight and stimulated the day after in complete growth medium, i.e., EMEM containing with 10% FBS and antibiotics. IL-6 release was measured with a specific ELISA assay (R&D Systems).

**Table 3:** Antibodies and reagents used for immunocytochemistry and flow cytometry

*Immunocytochemistry*		
mAbs or immunostaining reagents	Concentration	Final dilution used
DAPI (4′,6-diamidino-2-phenylindole, dihydrochloride) (ThermoFisher Cat. N. D1306)	5 mg/ml	1/2000
Rhodamine phalloidin (ThermoFisher Cat. N. R415)	200 U/ml	1/500
IBA1 (Abcam Cat. N. ab15690)	3.5 mg/ml	1/100
*Flow Cytometry*		
mAbs	Optimal Ab volume per test (1 × 10^6^)	Final dilution used
PE-conjugated anti-GFAP (BD Pharmingen Cat. N. 561,483)	5 μl	1/40
PE-conjugated anti-HLA-DR (BD Bioscience Cat. N. 562,304)	5 μl	1/50
FITC-conjugated anti-CD14 (BD Pharmingen Cat. n 555,397)	20 μl	1/10
PE-conjugated anti-CD11b (eBioscience™ Cat. N. 12–0118-42)	5 μl	1/20
PE-conjugated anti CD68 (BD Horizon Cat. N. 564,944)	5 μl	1/40
FITC-conjugated anti-HLA-ABC (BD Biosciences Cat. N. 555,552)	20 μl	1/10

**Table 4:** Sequences of specific primer sets, used for Q-PCR analysis

Genes	Forward primer	Reverse primer	Product length (Base pair)	NCBI Reference sequence	mRNA variants
Lineage markers					
IBA1	F150–170: TCATGTCCCTGAAACGAATG	R265–285: CCAGCATCATCCTGAGAAAG	136 bp	NM_032955.2	1, 3 and 4
CD14	F636–655: CGCTCGAGGACCTAAAGATA	R711–693: AAGCTGGAAAGTGCAAGTC	76 bp	NM_001174105.1	4
CX3CR1	F187-207: GGCTGAGGCCTGTTATATTG	R315–333: TGACACTCTTGGGCTTCT	147 bp	NM_001171174.1	1, 2, 3 and 4
CCR2	F425–445: CCACAAGCTGAACAGAGAAA	R558–579: GGGAGCACCGTAATCATAATC	155 bp	NM_001123041.2	A and B
P2RY12	F674–692: AGACCACCAGGCCATTTA	R837–858: CAGACTAGACCGAACTCTGAT	185 bp	NM_022788.4	1 and 2
CSF1R	F344–364: CAGGGAATCCCAGTGATAGA	R470–489: TGGAGCCATCAGAGTACAG	146 bp	NM_005211.3	2, 3 and 4
TMEM119	F408–428: ACTTCCTGGATGGGATAGTG	R542–562: GGGAAGGACGATGGGTAATA	155 bp	NM_181724.2	
TREM2	F588–607: AGCCTCTTGGAAGGAGAAA	R719–737: AGTTCACTGGGTGGATGT	150 bp	NM_018965.3	
Inflammatory genes					
IL-6	F394–414: CCTTCCAAAGATGGCTGAAA	R 524–543: TGGCTTGTTCCTCACTACT	150 bp	NM_000600.4	1 and 2
TNFα	F1305–1324: GAGCCAGCTCCCTCTATTTA	R 1485–1466: GGGAACAGCCTATTGTTCAG	181 bp	NM_000594.3	
IL-1β	F407-427: CATGGGATAACGAGGCTTATG	R 556–537: CCACTTGTTGCTCCATATCC	150 bp	NM_000576.2	
COX2 (Rat/ Human)	F1384–1407:TTGCTGGCAGGGTTGCTGGTGGTA	R1469–1449: CATCTGCCTGCTCTGGTCAATCGAA	86 bp	NM_000963.4	
NOS2 (Rat/ Human)	F 1701–1725: CTGCATGGAACAGTATAAGGCAAAC	R 1929–1905: AGACAGTTTCTGGTCGATGTCATGA	229 bp	AF049656.1	
TGFβ:	F2423-2442: CAGTCACCATAGCAACACTC	R 2583–2567: CCTGGCCTGAACTACTATCT	161 bp	NM_000660.5	
ARG1	F 905–925: GGGCTACTCTCAGGATTAGAT	R 1016–996: CGAAACAAGCCAAGGTTATTG	112 bp	NM_001244438.1	1 and 2
Normalization					
ACTB	F590–613: ACGTTGCTATCCAGGCTGTGCTAT	R830–807: TTAATGTCACGCACGATTTCCCGC	241 bp	NM_001101.3	

**Fig. 4:**
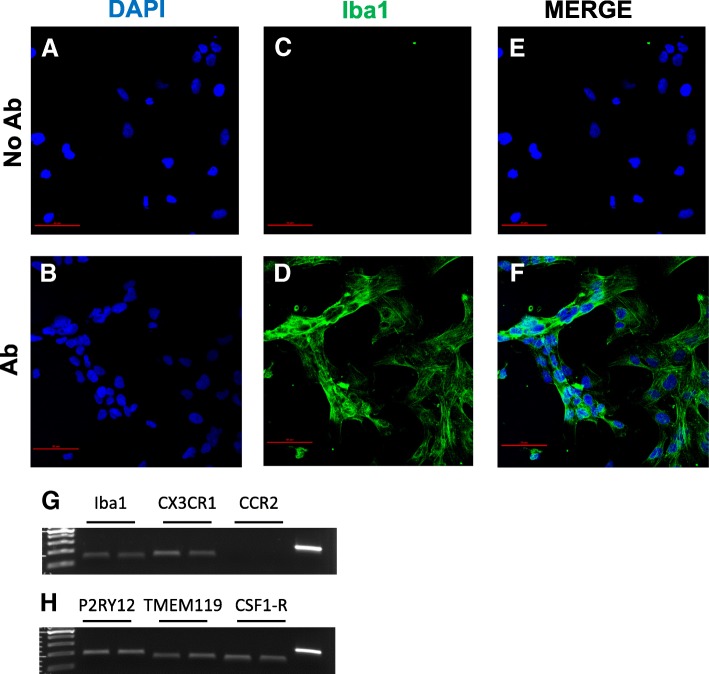
HMC3 cell expression of microglial lineage markers. **a**–**f** Representative example of confocal images (1024 × 1024 pixels) acquired at × 20 magnification with a confocal laser scanning system (A1+, Nikon). HMC-3 cells immuno-labeled for IBA1 (green fluorescence, **d**, **f**) and DAPI stained (blue fluorescence, **a**, **b**) are shown. Merged images are shown in (**e**, **f**). Control experiments performed by omitting the primary antibody are shown in (**a**, **c, e**). No green fluorescence was present (**c**, **e**), indicating neither spontaneous fluorescence nor non-specificity of the secondary antibody. Scale bar 50 μM. **g**–**h** Total RNA was prepared from human microglial HMC3 cells 24 h after incubation in complete growth medium and retrotranscribed using random hexamers. Real time (Q)-PCR analysis for the mRNA levels of IBA1, CX3CR1, CCR2, P2RY12, TMEM119, and CSF1-R was carried out according to our standard protocols [109]. **g**, **h** panels show a representative gel image of PCR products obtained at the end of the analysis from two different RNA samples for each condition. In the last line is shown the actin amplification, as positive control

**Fig. 5:**
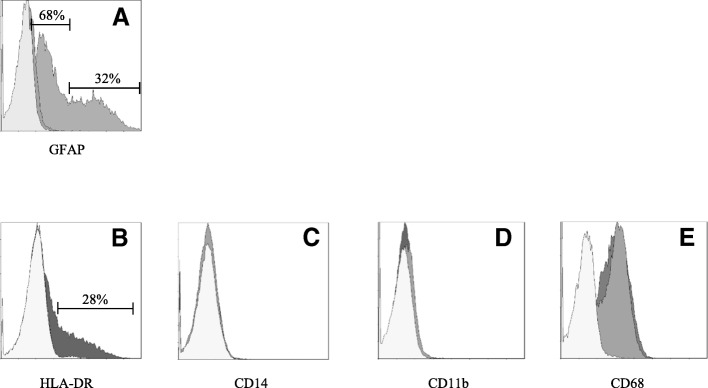
Phenotypical characterization of HMC3 cells under basal conditions and in response to IFNγ. HMC3 cells were plated at the density of 30,000 cells/cm^2^ in T25 flasks, and grown for 3 days when cells were almost confluent. In the experiments in which INFγ was used, cells were stimulated for the last 36 h. Controls did not receive any stimulus for the same time period (“resting” HMC3 cells). For detection of intracellular antigens, aliquots of 1 × 10^6^ cells in 100 μl were fixed using BD cytofix (BD Pharmingen) at 4 °C for 30 min, and permeabilized with BD FACS permeabilizing solution at 4 °C for 30 min. Cells were then stained using the following antibodies: PE-conjugated anti Human GFAP mouse Mab, BD Bioscience (**a**), and PE-CF594-conjugated anti Human CD68 mouse Mab, BD Bioscience (**e**), according to the manufacturer’s instructions. For the evaluation of surface antigens, aliquots of 5 × 10^5^ cells in 100 μl were directly incubated in PBS buffer containing the following antibodies: PE-CF594-conjugated anti Human HLA-DR mouse Mab, BD Bioscience (**b**); FITC-conjugated anti Human CD14 mouse Mab, BD Pharmingen (**c**); and PE-conjugated anti Human CD11b mouse Mab, E-Bioscience (**d**). Cells were analyzed by the 6-parameter (2 scatter and 4 fluorescence signals) Coulter Epics XL flow cytometer (Beckman-Coulter). Control histograms (white histograms) indicate level of cell autofluorescence in the emission wavelength that pertains the fluorochrome-conjugated Mab. Panel (**a**) shows expression of GFAP on HMC3 cells at passage 4 (dark gray), and on the human glioblastoma U373 cell line (light gray) that constitutively expresses GFAP. As autofluorescence signal in the two cell lines was similar, only one representative histogram is plotted in panel (**a**) (white histogram). As shown in this panel, the HMC3 cells stain negatively for GFAP (the dark gray histogram completely overlaps the background histogram), whereas the U373 cells express the target antigen. Two different populations expressing GFAP at different levels were identified (light gray plot): 68% of the U373 cells express GFAP at low level (GFAP-dim), whereas 32% of the cells express GFAP at higher level (GFAP-high). Panels (**b**–**e**) show results from HMC3 cells obtained at passage 7. In these panels, white histograms indicate cell autofluorescence, light gray histograms and dark gray histograms are representative of control (“resting”) HMC3 cells and IFNγ-treated HMC3 cells, respectively. Gating strategy: all analyses were obtained after gating according to morphological characteristics (not shown). As levels of autofluorescence did not change according to cell treatment, one representative example is plotted for each emission wavelength. Linear regions are used for calculating percentage of positive cells (plots A–B). Central tendency of CD68 expression on HMC3 cells in the different experimental conditions is represented by the mean fluorescence intensity (MFI, see text)

**Fig. 6:**
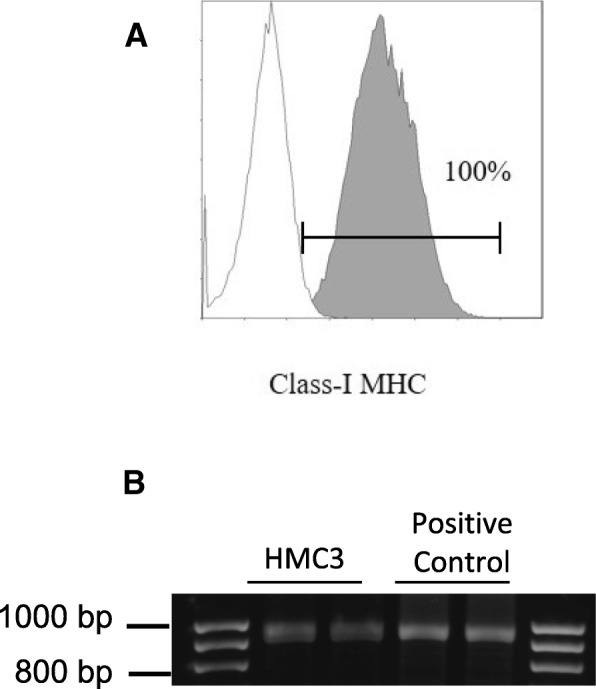
Expression of the human class I MHC antigens on HMC3 cells. **a** HMC3 were plated at the density 30,000 cells/cm^2^ in T25 flasks, and grown for 3 days when cells were almost confluent. For the detection of class I MHC surface antigens, aliquots of 5 × 10^5^ cells in 100 μl were directly incubated with FITC-conjugated anti human HLA-ABC mouse Mab, BD Bioscience. This antibody is specific for human MHCI antigens, and does not cross react with rat MHCI antigens. Cells were analyzed by the 6-parameter (2 scatter and 4 fluorescence signals) Coulter Epics XL flow cytometer (Beckman-Coulter). Results from HMC3 cells at passage 8 are shown. Gating strategy: the analysis was obtained after gating according to morphological characteristics (not shown). Background histogram (white) indicates level of cell autofluorescence. A linear region was used for calculating percentage of positive cells. **b** Gel image of PCR products obtained at the end of a HLA Locus B specific amplification protocol. The 922 bp amplicons were separated by electrophoresis through 1.5% agarose gels containing 0.1 μg/ml ethidium bromide. Lines 2–3 amplification products from genomic DNA extracted by HMC3 (ATCC®CRL-3304) cells; lines 4–5 amplification products from a positive control, i.e., an anonymous human genomic DNA sample provided by ViiV Healthcare Ldt

The morphology of a viable culture is shown in Fig. 3, i.e., 1 day after plating (Fig. 3a) and when cells reached confluency (day 3 post plating, Fig. 3b). In line with Etemad and colleagues [29], the HMC3 (ATCC®CRL-3304) cells display a complex morphology, with both globular, bipolar, and elongated cells detected in culture by rhodamine-phalloidin staining (Fig. 3c–e). We confirmed that cells are IBA1 positive (Fig. 4d–g). Moreover, we detected at the mRNA level the expression of CX3CR1 and CSF1-R (Fig. 4g–h), together with other specific markers, comprised in the recently characterized microglial signature [2], i.e., P2RY12 and TMEM119 (Fig. 4h). The CCR2 transcript was undetectable (Fig. 4g), in line with previous observations [29, 38]. Consistently with the literature and the ATCC®‘s data sheet, the HMC3 cells were GFAP negative (Fig. 5a, i.e., the dark gray histogram completely overlaps the background histogram). The glioblastoma U373 cell line, kindly provided by Prof. Grassi (Institute of Physiology, Catholic University Medical School, Rome, Italy), was used as a positive control for the anti-GFAP antibody (Fig. 3a). The activation marker MHCII (HLA-DR in the figure) was negative under basal condition and upregulated in the 28% of the HMC3 cell population by IFNγ treatment (36 h, 10 ng/ml) (Fig. 5b). In addition, resting HMC3 cells were CD14 (Fig. 5c) and CD11b (Fig. 5d) negative, and these markers were not induced by IFNγ. With respect to CD14 expression, our results are in agreement with the original characterization [17] and the evaluation performed in flow cytometry by Etemad and colleagues [29]. Finally, the HMC3 (ATCC®CRL-3304) cells expressed CD68 under basal conditions (Fig. 5e), in agreement with previous observations [17, 29]. However, CD68 was expressed at low level indicated by a mean fluorescence intensity (MFI) of 3.3, and no further induction with IFNγ was detected (MFI = 3.6). As shown in Fig. 6a, the HMC3 (ATCC®CRL-3304) cells were found entirely and homogenously positive for human leukocyte antigens A, B, and C (MHCI antigens). The antibody used for this analysis is specific for the human antigens and does not cross react with rat antigens. In addition, a HLA-B-specific amplification between the first and the third intron was carried out by PCR on genomic DNA samples, extracted by the HMC3 (ATCC®CRL-3304) cells using the QIAamp® DNA Blood mini kit (Qiagen, Hilden, Germany) [110]. The HLA-B locus-specific amplification was performed using the following validated primers: 5BIn1–57 forward primer (forward 5´-GGGAGGAGCGAGGGGACCG/CCA G-3′; intron 1 36–57) and 3BIn3–37 reverse primer (reverse 5´-GGAGGC ATCCCCGGCGACCTAT-3′; intron 3 37–59), yielding a 922 bp product [111]. The PCR reaction contained 100 ng total DNA, 1X PCR buffer, 300 nM of each primer, 1.25 U AccuPrime™ PfX DNA Polymerase (Invitrogen Corporation) in 50 μl final volume. After initial denaturation (10 min at 95 °C), a total of 40 PCR cycles were conducted, using the following two-step PCR conditions: denaturation at 95 °C for 20 s and annealing/extension at 68 °C for 1 min, in a MasterCycler Ep thermocycler® (Eppendorf, Hamburg, Germany). The amplicons were separated by electrophoresis through 1.5% agarose gels containing 0.1 μg/ml ethidium bromide. A human genomic DNA sample was provided by ViiV Healthcare Ldt. and used as positive Control for the amplification [112]. Data are presented in Fig. 6b. Taken together, this evidence further confirm the human origin of the HMC3 (ATCC®CRL-3304) cell line.

Consistently with the literature, the HMC3 (ATCC®CRL-3304) cells produced sizable amounts of ROS. Reactive free radicals were measured using H2DCF-DA [2,7- dichlorodihydrofluorescein diacetate (Invitrogen)]. At the end of the experiment, the incubation medium was replaced by Hank’s balanced salt solution (HBSS) containing 20 μM of H2DCF-DA [20]. Cells were incubated at 37 °C, in a humidified atmosphere with 5% CO_2_, for 45 min. The fluorescence signal due to H2DCF-DA oxidation within the cells was quantified using a microplate fluorescence reader (PerkinElmer Inc.), using 485 nm as excitation and 535 nm as emission wavelength [113]. Pro-inflammatory cytokines per se, at 10 ng/ml, did not modify basal levels of ROS production, whereas IL-1β and TNFα each used in combination with IFNγ slightly increased ROS production by HMC3 cells (0.1–0.2-fold increases), in line with previous observations [21]. However, this effect was not consistent among different experiments, carried out between passage 6 and 13. Interestingly, the production of ROS by HMC3 cells growth in either MEM or DMEMF12 was significantly lower, thus suggesting a less activated phenotype under basal conditions when kept in these media (not shown). In addition, we assessed the production of NO indirectly by measuring nitrite accumulation in the incubation media [108]. Nitrite levels were undetectable under basal conditions and remained so after a 24-h treatment with different pro-inflammatory stimuli, including LPS, LPS/IFNγ, and IL-1β, and TNFα used alone or in combination with IFNγ (data not shown). Consistently, resting HMC3 (ATCC®CRL-3304) cells did not express iNOS, neither its expression was induced by pro-inflammatory activation (Table 5). Conversely, we observed a basal expression of IL-6 (Table 5) in resting HMC3 cells, together with a sizable release of the cytokine in the incubation medium under basal conditions, i.e., 150 pg/ml. The expression level of IL-6 increased in response to pro-inflammatory cytokines, particularly IL-1β in combination with IFNγ (II, Table 5). In addition, the cytokine mixture (II) stimulated basal IL-6 secretion by 20-folds (data not shown). Other pro- and anti-inflammatory genes were studied at transcriptional levels under basal conditions and after 24-h treatments with II (Table 5). The cytokine mixture promoted a significant up-regulation of IL-1β, TNFα, and COX2, leaving unmodified the expression of TGFβ and ARG1. In line with our data, it has been recently shown that the HMC3 (ATCC®CRL-3304) cells express at the mRNA level several pro-inflammatory genes, including IL-1β, TNFα, IL-6, COX2, and in addition heme oxygenase-1 [31]. Interestingly, lumirubin, a photoisomer of bilirubin (generated during phototherapic treatment of neonatal hyperbilirubinemia), promoted microglial inflammatory activation as indicated by the upregulation of all these genes except COX2 [31]. Moreover, the pro-inflammatory effects of bilirubin photoisomers were more prominent on rat hippocampal slices, further highlighting relevant differences between human and rodent experimental models. Finally, increased expression and release of both IL-1β and IL-18 has been observed in the HMC3 (ATCC®CRL-3304) cells by treatment with 100 ng/ml LPS for 24 h followed by additional 30 min with 5 mM adenosine triphosphate (ATP) [32]. Exposure of HMC3 (ATCC®CRL-3304) cells to LPS/ATP was associated to increased expression of the intracellular NOD-like receptor 3 and activation of caspase 1, together with a significant increase of cFos nuclear protein levels. Pro-inflammatory effects of LPS/ATP were indeed reversed by knockdown of cFos. [32]. The treatment with LPS/ATP significantly affected microglial viability. All the effects of the LPS/ATP combination were reduced by the α-2 adrenergic agonist, dexmedetomidine [32]. In these research papers, the HMC3 cells were maintained in MEM [31] or EMEM [32] with 10% FCS and antibiotics.

**Table 5:** Gene expression analysis of the HMC3 (ATCC®CRL-3304) cells

Gene	Control (mean threshold cycle, Ct)	II (Fold variation relative to control)
Pro-inflammatory genes		
IL-6	27.97 ± 0.17 (*n* = 12)	43.75 ± 2.59 (*n* = 11)
TNFα	32.50 ± 0.20 (*n* = 12)	5.37 ± 0.45 (*n* = 11)
IL1β	29.02 ± 0.10 (*n* = 12)	4.71 ± 0.32 (*n* = 12)
COX2	33.00 ± 0.50 (*n* = 9)	2.51 ± 0.46 (*n* = 9)
NOS2	No Ct (*n* = 9)	No Ct (*n* = 9)
Anti-inflammatory genes		
TGFβ	28.50 ± 0.16 (*n* = 12)	1.02 ± 0.06 (*n* = 12)
ARG1	29.51 ± 0.10 (*n* = 10)	1.07 ± 0.07 (*n* = 11)

## Conclusions

Evidence reviewed in the present paper suggest that the human microglial cell line clone 3 generated in the laboratory of Prof. Tardieu has been extensively characterized, with respect to cell morphology, antigenic profile, and cell functions. However, cells were used under different denominations, i.e., CHME3, HMC3, C13-NJ, and CHME-5, which contributed to data fragmentation. In this regard, the experimental data reviewed in this article show that the cells, regardless the name adopted, retain a similar antigenic profile together with similar functional properties. The cells are capable of responding to a pattern of chemokines and inflammatory stimuli, regulating the expression of typical activation markers of microglia. Therefore, these cell lines should be regarded as a unique experimental model. Caution should be taken with data obtained using microglial cells named CHME-5, since it has been reported that several aliquots in use in different laboratories were cross contaminated by rat glioma cells [19]. Laboratories using the CHME-5 cells should perform an authentication procedure to confirm the human origin. In this regard, the HMC3 (ATCC®CRL-3304) cells present the advantage to have been authenticated by ATCC® according to a complex procedure, including morphology evaluation, karyotyping, and PCR based approaches to confirm the identity of human cell lines and to rule out both intra- and inter-species contamination. In addition, we showed that the HMC3 (ATCC®CRL-3304) cells retain most of the original antigenic properties and express also more specific microglial markers (recently identified) [2]. Resting cells produce significant amounts of ROS and IL-6. The latter can be further induced by pro-inflammatory cytokines (particularly IL-1β in combination with IFNγ at 10 ng/ml). Evidence from the literature together with our original data may represent an important starting point for researchers that are planning to use this experimental model.

## Abbreviations

ACTB: β-actin
ADAMTS: A disintegrin and metalloproteinase with thrombospondin motifs
APP: Amyloid beta precursor protein
ARG: Arginase
ATCC®: American Type Culture Collection
ATP: Adenosine triphosphate
Aβ: Amyloid beta peptide
BDNF: Brain derived neurotrophic factor
CCL: C-C motif chemokine ligand
CCR: C-C chemokine receptor
CD: Cluster of differentiation
CHME3: Human microglial clone 3 cell line
CM: Conditioned medium
CNS: Central nervous system
coMTb: Human peripheral blood monocytes infected with *Mycobacterium tuberculosis*
COX: Cyclooxygenase
CSF1-R: Colony-stimulating factor 1 receptor
CXCR: C-X-C chemokine receptor
DAPI: 4′,6-Diamidino-2-phenylindole dihydrochloride
DHA: Docosahexaenoic acid
DMEM: Dulbecco’s modified minimal essential medium
ELISA: Enzyme-linked immunosorbent assay
EMEM: Eagle’s minimum essential medium
EPA: Eicosapentenoic acid
ERK: Extracellular signal–regulated kinase
FCS: Fetal calf serum
FITC-conjugated: Fluorescein isothiocyanate conjugated
GFAP: Glial fibrillary acidic protein
GM-CSF: Granulocyte-macrophage colony-stimulating factor
H2DCF-DA: 2,7-Dichlorodihydrofluorescein diacetate
HBSS: Hank’s balanced salt solution
HCV: Hepatitis virus C
HIV: Human immunodeficiency virus
HLA-DR: Human leukocyte Antigen D Related
HMC3: Human microglial clone 3 cell line
hTERT: Human telomerase reverse transcriptase
IBA1: Ionized calcium binding adaptor molecule 1
IFNγ: Interferon-γ
II: Interferon-γ/interleukin-1β
IL: Interleukin
iNOS: Inducible nitric oxide synthase
IP-10: Interferon-γ inducible protein 10
IRES: Internal ribosome entry site
IRF: Interferon regulatory factor
JAK: Janus kinases
JEV: Japanese encephalitis virus
LOX: Lipoxygenase
LPS: Lipopolysaccharide
LXA4: Lipoxin A4
Mab: Monoclonal antibody
MAPK: Mitogen-activated protein kinase
MaR: Maresin
MCP-1: Monocyte chemoattractant protein 1
M-CSF: Macrophage colony-stimulating factor
M-CSF-R: Macrophage colony-stimulating factor receptor
MFI: Mean fluorescence intensity
MHCII: Class II major histocompatibility complex
miR- or miRNA: MicroRNA
MMF: Monomethylfumarate
MMP: Matrix metalloprotease
NF70KD: Neuronal neurofilament staining
NFκB: Nuclear factor kappa-light-chain-enhancer of activated B cells
NGF: Nerve growth factor
NO: Nitric oxide
NOS2: Inducible nitric oxide synthase
NOX: NADPH oxidase
NPCs: Neuronal precursor cells
NS3: Nonstructural protein 3, also known as p-70
NSE: Nonspecific esterase activity
P2RY12: Purinergic receptor 12
PE-conjugated: Phycoerythrin-conjugated
PSA-NCAM: Polysialylated neural cell adhesion molecule
RANTES: Regulated on activation, normal T cell expressed and secreted
ROS: Free oxygen radicals
RvD1: Resolving D1
sAPPα: Secreted amyloid precursor protein-α
SH_swe_: Human neuroblastoma SH-SY5Y cells overexpressing APP_695_ Swedish mutation protein
STAT: Signal transducer and activator of transcription
SV40: Simian vacuolating virus 40
TGF: Transforming growth factor
TIMP: Tissue inhibitor of metalloproteinases
TLRs: Toll-like receptors
TMEM119: Transmembrane protein 119
TNFα: Tumor necrosis factor α
TRAF: TNF receptor-associated factor
TREM2: Triggering receptor expressed on myeloid cells 2
TRIM: Tripartite motif-containing protein
TRITC: Tetramethylrhodamine
UTR: Untranslated regions
VEGF: Vascular endothelial growth factor
α7nAcR: α7 nicotinic acetylcholine receptor
αMSH: α-melanocyte stimulating hormone
